# Experiences and perceived determinants of healthcare-related time toxicity among women with gestational diabetes mellitus: a qualitative study

**DOI:** 10.3389/fendo.2026.1947845

**Published:** 2026-09-24

**Authors:** Jingru Liu, Wei Xu, Jing Ma, Xueying Shang, Mengmeng Jia, Yanli You

**Affiliations:** 1 Department of Gastrointestinal Surgery, The First Affiliated Hospital, and College of Clinical Medicine of Henan University of Science and Technology, Luoyang, China; 2 School of Nursing, Henan University of Science and Technology, Luoyang, China; 3 Department of Obstetrics, The First Affiliated Hospital, and College of Clinical Medicine of Henan University of Science and Technology, Luoyang, China; 4 Department of Nursing, The First Affiliated Hospital, and College of Clinical Medicine of Henan University of Science and Technology, Luoyang, China

**Keywords:** antenatal care, gestational diabetes mellitus, nursing support, qualitative study, self-management, time toxicity

## Abstract

**Background:**

Gestational diabetes mellitus (GDM) requires women to attend follow-up visits and complete repeated self-management tasks during pregnancy. These tasks include blood glucose monitoring, meal planning, physical activity, health education and medication when needed. They may create a time-related burden in daily life. However, little is known about women’s experiences of healthcare-related time toxicity during GDM management. This study aimed to explore women’s experiences of healthcare-related time toxicity and the perceived factors shaping these experiences.

**Methods:**

A qualitative descriptive study was conducted in the obstetric outpatient clinics of a tertiary teaching hospital in Henan Province, central China. Women with GDM were recruited using purposive sampling. Individual semi-structured interviews were conducted with 13 participants. The interviews were audio-recorded, transcribed verbatim and analysed using qualitative content analysis.

**Results:**

Four themes and 13 subthemes were generated from the interview data: (1) GDM added a new timetable to pregnancy; (2) hospital follow-up made time costs visible; (3) work and family roles compressed time for GDM management; and (4) women needed support that reduced time and decision burden.

**Conclusion:**

Healthcare-related time toxicity in GDM was experienced as both visible hospital time and hidden daily self-management work. Women’s time burden was not produced by GDM tasks alone. The organisation of services, family caregiving, work schedule, and outpatient processes also play a role. There is a need for the nurse, midwife, and/or diabetes educator to be specific and user-friendly in their support, such as providing clear examples of diet, review of blood glucose levels online in a timely fashion, and improved coordination of prenatal, nutrition and blood glucose-related appointments. These changes may decrease unnecessary traveling, waiting, and redundant decision-making, and assist women to incorporate the management of GDM into their daily lives more successfully.

## 1 Introduction

Gestational diabetes mellitus (GDM) is defined as hyperglycaemia that is first identified during pregnancy. This is a common metabolic problem during pregnancy. The International Diabetes and Pregnancy Study Group estimated that the prevalence of GDM is about 14.0% worldwide (1). In mainland China, the pooled prevalence has been reported as 14.8% (95% CI: 12.8%–16.7%) (2). GDM is associated with gestational hypertension, caesarean delivery, large-for-gestational-age infants, neonatal hypoglycaemia and long-term metabolic risks for both mothers and offspring (3). Therefore, early identification and standardised management remain important parts of antenatal care.

GDM management usually includes dietary control, physical activity, blood glucose monitoring, health education and medication when needed (3). In China, women with GDM are often managed through outpatient follow-up, health education, dietary guidance and blood glucose monitoring (4). These strategies can improve glycaemic control and reduce adverse outcomes. However, they also require women to take on repeated care tasks during pregnancy. After diagnosis, women need to adjust their daily routines. They may need to monitor blood glucose several times a day, record glucose values, plan meals, arrange activity after meals and attend follow-up visits. These tasks continue until delivery. They also occur alongside work, housework, childcare and pregnancy-related discomfort. Thus, GDM management can impose practical and emotional strain on daily life.

Several studies have investigated women’s self-management experiences after GDM diagnosis. A systematic review identified obstacles related to diet, exercise, glucose monitoring, health education, family support, and social context (5). Another recent qualitative study also reported psychosocial challenges among women with diabetes in pregnancy, including distress at diagnosis, dietary guilt, role conflict, and concerns about insulin use (6). These studies show that GDM management is not only a clinical issue. It is also shaped by women’s everyday circumstances.

In recent years, the term time toxicity has been used in cancer care. It refers to the burden of time spent travelling, waiting, receiving treatment, attending follow-up and managing care-related tasks at home (7). Although time toxicity has been studied predominantly in oncology, it is increasingly being investigated across various medical disciplines, while evidence in obstetric care remains limited (8). A qualitative study further showed that time burden may include both visible time in healthcare settings and less visible tasks outside hospital (9). In GDM care, similar time costs may occur through repeated outpatient visits, waiting, glucose monitoring, dietary planning, postprandial activity and communication with healthcare professionals. However, this aspect has received limited attention in previous GDM studies.

To our knowledge, few studies have explored GDM management from the perspective of time toxicity. Existing studies have mainly focused on self-management behaviours, psychological experiences, educational needs and social support (6, 7). However, women’s experiences of time spent on GDM-related care are still not well understood. Therefore, this study aimed to explore women’s experiences of healthcare-related time toxicity during GDM management and the factors influencing these experiences. The findings may help nurses, midwives and diabetes educators understand the time-related difficulties faced by women with GDM, and provide guidance for developing targeted support strategies, improving follow-up arrangements and promoting more individualized GDM care.

## 2 Methods

### 2.1 Study design

A qualitative descriptive design was chosen because the study sought a direct account of women’s experiences and perceived influencing factors while remaining close to participants’ own descriptions, rather than developing a new theory or an interpretive account of the phenomenon (10). The study explored women’s experiences of healthcare-related time toxicity during gestational diabetes mellitus (GDM) management and the perceived factors shaping these experiences. The study was conducted in the obstetric outpatient clinics of a large tertiary teaching hospital in Henan Province, central China. The reporting of this study followed the Consolidated Criteria for Reporting Qualitative Research (COREQ) checklist (11).

### 2.2 Sampling and recruitment

Participants were recruited from obstetric and perinatal outpatient clinics between January and December 2023. Clinicians briefly introduced the study to potentially eligible women during their outpatient visits. Women who expressed interest were referred to the research team, after which a researcher confirmed their eligibility, provided detailed information about the study, and obtained written informed consent. Purposive sampling was used. Maximum variation was considered in age, gestational age, parity, occupation, education level, insulin use and family history of diabetes.

The inclusion criteria were: (1) women diagnosed with GDM based on a 75-g oral glucose tolerance test; (2) women receiving regular antenatal care at the study site; (3) women able to communicate in Mandarin; and (4) women willing to share their experiences and provide written informed consent. Women were excluded if they had pregestational diabetes, cognitive impairment, severe psychiatric disease, communication difficulty, or a physical condition that made an interview unsuitable.

### 2.3 Data collection

Semi-structured, one-to-one interviews were conducted in Mandarin. Two trained researchers conducted the interviews. They had received unified training in qualitative interviewing.

The interview guide was developed after a literature review and group discussion. It was reviewed by three experts in obstetric nursing, diabetes care and qualitative research. Women with GDM were not formally involved in co-designing the interview guide. Two pilot interviews were conducted to test the clarity and order of the questions. The pilot data were not included in the analysis. The main interview questions are shown in **Table 1**. The technical term ‘time toxicity’ was not used directly in the interview questions. Instead, participants were asked in everyday Mandarin about time spent on GDM-related care, changes in daily routines, and the practical and emotional effects of these time demands. This wording was used to reduce conceptual priming and possible misunderstanding of the term ‘toxicity’.

**Table 1:** Outline of the semi-structured in-depth interview.

Number	Main focus
1	Please describe what a usual day was like before you were diagnosed with GDM.
2	How did you learn that you had GDM? What were your thoughts and feelings at that time?
3	What changes occurred in your daily rhythm after the diagnosis?
4	Which parts of GDM management took the most time?
5	How did healthcare visits, examinations and self-management affect your family life or work life?
6	What feelings did you usually have when spending time on diet control, blood glucose monitoring, physical activity, insulin injection if applicable, or follow-up visits?
7	What made GDM management more difficult? What made it easier?
8	Looking back on this experience, what was the greatest impact of GDM on your life? What support did you expect from healthcare professionals?

Before each interview, the researcher explained the purpose and process of the study. Written informed consent and permission for audio recording were obtained. Interviews were conducted in a quiet room in the outpatient clinic or in another private place chosen by the participant. Only the participant and interviewer were present. Each participant was interviewed once. The interviewers had no prior relationship with the participants before recruitment. Interviews lasted 25–40 min. Field notes were taken during and immediately after each interview. A short questionnaire was used to collect demographic and clinical information after the interview. Participants were anonymised as P1-P13 in transcripts, tables and quotations.

Data collection and analysis were conducted concurrently. After each interview, the research team reviewed the emerging codes and categories to determine whether additional information relevant to the research aim had been identified. Recruitment continued until data saturation was reached. Saturation was considered achieved when no new codes, categories, or relevant information emerged from the later interviews, resulting in a final sample of 13 participants (12).

### 2.4 Data analysis

Data analysis was conducted at the same time as data collection. Audio recordings were transcribed verbatim in Chinese within 24 h after each interview. The transcripts were checked against the recordings by another researcher. NVivo 15 software was used to manage the transcripts, codes and categories.

The transcripts were analysed using content analysis (13). First, two researchers read each transcript several times to become familiar with the data. Second, meaningful units related to time toxicity and its perceived determinants were identified. Third, the meaningful units were coded line by line. Fourth, similar codes were grouped into categories. Fifth, categories were compared and refined into subthemes and themes. The analysis focused on both women’s experiences and the factors that made GDM management more difficult or easier.

Two researchers coded the data independently at the early stage. They then compared codes and discussed differences. Disagreements were resolved through team discussion. Preliminary themes were reviewed by the research team. Representative quotations were selected to support each theme. Chinese quotations were translated into English by one bilingual researcher and checked by another researcher to preserve the original meaning.

### 2.5 Ethical considerations

This study was approved by the institutional ethics committee (No. 2022-0334). All participants received information about the study aim, procedures, voluntary participation and confidentiality. They were informed that they could withdraw at any time without affecting their medical care. Written informed consent was obtained before the interview. All audio recordings, transcripts and field notes were stored on password-protected computers. Personal identifiers were removed from the transcripts. Participant codes were used in all tables and quotations.

### 2.6 Rigor

Several strategies were used to enhance the rigor of the qualitative findings. Credibility was supported by in-depth interviews, field notes and team discussion. As part of member checking, a summary of the preliminary findings was returned to three participants to determine whether the findings adequately reflected their experiences. Their feedback was used to assess and enhance the credibility of the findings. Dependability was supported by an audit trail that recorded recruitment, interview, transcription and coding decisions. Confirmability was enhanced by reflexive notes. Researchers recorded their assumptions about GDM care, time burden and patient self-management during analysis. Transferability was supported by describing the study setting, sampling process, participant characteristics and interview context in detail (14).

## 3 Results

### 3.1 Participant characteristics

A total of 13 women with GDM participated in the interviews. Their ages ranged from 23 to 38 years. At the time of the interviews, their gestational ages ranged from 28 to 37 weeks. Participant information and pregnancy-related information are shown in **Table 2**. Seven women were in their first pregnancy, and six reported two pregnancies including the current pregnancy. Ten women had no previous delivery, and three had one previous delivery. Three women used insulin during pregnancy. Four women reported a family history of diabetes.

**Table 2:** Participant information at the time of the interview.

No.	Participant information				Pregnancy-related information			
	Age (years)	Occupation	Education level	Family history of diabetes	Gestational age (weeks)	Pregnancy count	Existing children, n	Insulin use
P1	27	Nurse	Bachelor	No	36	2	0	No
P2	31	Office worker	Bachelor	No	35	2	0	No
P3	34	Office worker	Junior college	No	33	1	0	No
P4	25	Office worker	Junior college	Yes	28	1	0	No
P5	35	Military personnel	Bachelor	No	32	2	1	No
P6	29	Physician	Master	No	33	2	1	No
P7	28	Homemaker	Junior college	Yes	33	1	0	Yes
P8	23	Homemaker	High school	No	35	1	0	No
P9	24	Farmer	High school	No	35	1	0	No
P10	32	Civil servant	Bachelor	No	33	1	0	No
P11	27	Athlete	Bachelor	Yes	34	2	0	Yes
P12	28	Teacher	Bachelor	Yes	34	1	0	Yes
P13	38	Self-employed	Bachelor	No	37	2	1	No

Four themes and 13 subthemes were generated from the interview data. The first two themes described women’s experiences of time toxicity. The last two themes reflected perceived factors that intensified or alleviated these experiences. **Table 3** summarises the themes and subthemes.

**Table 3:** Themes and subthemes.

Theme	Subthemes
GDM added a new timetable to pregnancy	A diagnosis introduced extra glucose tasks Monitoring and recording divided the day Diet, activity and insulin required repeated planning Continuous attention to glucose reduced freedom
Hospital follow-up made time costs visible	Long waits for brief consultations Travel and late pregnancy made visits physically tiring Fragmented procedures increased coordination work
Work and family roles compressed time for GDM management	Work schedules conflicted with recommended timing Childcare and housework competed with self-care Family support saved time but sometimes felt like supervision
Women needed support that reduced time and decision burden	Specific guidance reduced searching and guessing Online feedback reduced unnecessary visits Integrated outpatient arrangements reduced repeated waiting and travel

### 3.2 GDM added a new timetable to pregnancy

Following diagnosis, participants perceived pregnancy as more structured. They continued to balance work, family responsibilities, and physical discomforts. Simultaneously, they took on glucose monitoring, meal planning, postprandial activity, clinic visits, and (for some) insulin use. Many women characterised this as an extra timetable or a set of daily tasks.

#### 3.2.1 A diagnosis introduced extra glucose tasks

Following the diagnosis, participants often felt shocked and worried about the foetus. Nevertheless, the principal time-related change was practical in nature. Women had to rebuild their daily schedules around blood glucose control. Pregnancy thus shifted from a naturally unfolding experience to a routine organised around tasks.

“My day used to be arranged around work and rest. After diagnosis, it felt as if there was an extra set of blood glucose tasks. (P1)”

“I felt that I was not living a normal pregnancy. I was living according to a glucose management table. (P7)”

#### 3.2.2 Monitoring and recording divided the day

Blood glucose monitoring was short each time. The primary difficulty was timing. Women must be mindful of the fasting time, note the time of the last meal, and take the post-meal tests within the designated period. When they miss this time, they fear that the records will not show what their true glucose control is.

“In the morning I checked my fasting glucose, then I had to remember the time and test it again. (P3)”

“I activated an alarm on my phone, and I got a sense throughout the day that the glucose was calling my name, if you will, throughout the day. (P8)”

“Well, if I do not do the testing during the two hours after I eat, I’m not very happy. I don’t think the records on that day will be very useful. (P4)”

#### 3.2.3 Diet, activity and insulin required repeated planning

The control of diet is said to be a daily habit, not just a diet. Women have to constantly think about what to choose for staple food, if they should include or leave out fruits, how much oil to use, and if they feel full after eating. This is a diurnal schedule which is complicated even further by post-meal activities and insulin administration. All activities related to eating, walking, injection, and monitoring must be made at one time, leaving virtually no scope for spontaneous changes.

“Now eating is something I have to count on. Even if it’s just a little piece of fruit – I can’t relax. (P2)”

“I spend a lot of time trying to order the right food when I eat out, as I don’t always know what I can eat or what will impact my glucose. (P4)”

“Once I begin taking insulin, I must remember when to take it, the dosage and when to eat. (P11)”

#### 3.2.4 Continuous attention to glucose reduced freedom

Some women think that GDM has absorbed too much of their attention and time has been used up in completing particular tasks. They are nervous before the tests and look for explanations for the high glucose levels. With repeated attention to glucose levels, over time the woman gets weary, irritated, and feels the pregnancy is no longer free as it was before.

“I get nervous before every glucose test, particularly before I eat something I don’t know or some takeout food. (P2)”

“Not only am I physically tired; I always think about this topic repeatedly. (P10)”

*“*GDM has consumed lots of my time and it has impacted my mood as well*. (P7)”*

### 3.3 Hospital follow-up made time costs visible

It is not a straightforward cost of time to describe for the participants the time in the hospital. Visits to the hospital take a certain amount of time. Women must travel, register, line up for a series of exams, wait for results, and then go to a quick consultation. This is more challenging for working women, women who live far from the hospital, and women who are in late pregnancy.

#### 3.3.1 Long waits for brief consultations

Several participants made comparisons between the quick consultations and the longer ones which ensued afterward. If the visits were primarily about checking the glucose record or providing regular guidance, then the waiting time and movement around in the hospital was felt to be too much.

“Sometimes I spend a whole day to have just a few minutes with the doctor. (P1)”

“As the exam is only for a few minutes, but the time taken for registration, waiting in line, getting checked, and waiting for the result is a lot. (P2)”

#### 3.3.2 Travel and late pregnancy made visits physically tiring

The burden of follow-up also occurred in the body. Long journeys, queues, walking between departments and waiting became harder as pregnancy advanced. For these women, hospital attendance meant both lost time and physical fatigue.

“My home is far from the hospital. Every check-up has to be planned in advance. It takes time to travel, and after I arrive, I still have to queue and wait for the doctor to review the results. (P9)”

“Now I am in late pregnancy. Travelling to the hospital, queuing and waiting for examinations all make me tired. (P8)”

#### 3.3.3 Fragmented procedures increased coordination work

During these visits, some women also took time to prepare for the visit. They would need to seek permission, make childcare arrangements and/or organise transport. If antenatal, nutritional and glucose reviews were scheduled individually, the visit became more fragmented and needed repeated coordination.

“When they’re not done on the same day, I end up having to run back and forth, and it’s hard when I’m tired as a pregnant woman. (P2)”

“There must be work coordination for each visit; I can’t just go out and leave right away. (P5)”

### 3.4 Work and family roles compressed time for GDM management

Participants learned to cope with GDM at the level of their work and family life. These roles limit the time available for diet control, activities, glucose monitoring and follow-up. The challenges are not just about individual motivation; in many cases the management of GDM needs to be at odds with the realities of work, parenting and housekeeping.

#### 3.4.1 Work schedules conflicted with recommended timing

The suggested schedules have proven challenging for women working in the medical field, in the classroom, at the desk, and in other professions. Rests are not always possible for meals, glucose checks, and post-meals walks. These situations demonstrated that there are many times when the recommendations are not followed due to the structure of the work, not lack of willingness to follow the recommendations.

“While working, it is difficult to check glucose on time if one is seeing patients or taking over the shift. (P1)”

“It is impossible to me as a doctor to simply halt the work since I need to eat or measure my glucose. (P6)”

“If the two-hour post-meal monitoring period falls during class, as a teacher, I will not be able to stop to monitor my glucose mid-class. (P12)”

#### 3.4.2 Childcare and housework competed with self-care

The second layer of time pressure was described as being an extra burden on women who have already had children. The suggested time to take post-meal walks or blood glucose checks can sometimes fall during activities like childcare, cooking or cleaning, which can make it easy for them to have their self-management time disrupted.

“Went out for a walk afterward, but sometimes the older child asked to stay with him and play with him after the meal. (P13)”

“I have to work, and I have an older child to take care of, so I can’t spend all of my time on myself. (P5)”

#### 3.4.3 Family support saved time but sometimes felt like supervision

Familiar people are willing to help by cooking appropriate foods, going with the participants, or reminding them to check their glucose. This kind of support can alleviate the actual burden of work. But sometimes reminders are frequent enough to make women feel watched or judged by the family members when they meant to be helping.

“I would feel it would be easier if my family prepared appropriate food to eat for me and walked around with me after eating. (P8)”

“With all the family members asking me if I can have this or that, I get stressed sometimes, but they know how to do that. (P7)”

### 3.5 Women needed support that reduced time and decision burden

It wasn’t just that participants wanted more information. They aim to have support measures that can be implemented in everyday life and minimise the time of unnecessary hospitalisation. Suggestions they give are largely directed towards specific dietary advice, timely feedback on the internet, and improved coordination of outpatient care.

#### 3.5.1 Specific guidance reduced searching and guessing

Typical diet, exercise, and blood sugar monitoring recommendations are too vague. Women would like more specific information, such as meal combinations, cafeteria choices, takeout options, how much fruit to eat, insulin injection times, and what to do if they do not take a blood sugar test. Specific suggestions can help decrease the search engine volume and the number of times uncertainty is caused.

“I hope that doctors and nurses can simplify things a bit, like what to eat for each meal, how to eat fruits, what to do when blood sugar is high. (P8)”

“If someone could tell me how to combine my diet, I wouldn’t have to worry about it every day. (P4)”

“Even as a doctor, I still hope to receive practical advice based on my own work, such as how to arrange snacks when busy, or what to do if I miss the post-meal test. (P6)”

#### 3.5.2 Online feedback reduced unnecessary visits

Online review was seen as useful when the problem was limited to glucose records, diet adjustment or a simple question. Women felt that remote feedback could save travel, waiting and leave from work. They also believed that it should not become another source of frequent reminders.

“If there was online follow-up and I could send my glucose record to the doctor or nurse, it could reduce some unnecessary offline visits and save a lot of time. (P10)”

“Sometimes I only wanted to ask whether a glucose value mattered or whether I could eat a certain food. If I had to go to hospital every time, it was too much trouble. (P9)”

“Online follow-up is helpful, but it should not become another burden. If there is too much information and too many reminders, I would feel more pressure. (P1)”

#### 3.5.3 Integrated outpatient arrangements reduced repeated waiting and travel

Women hoped that antenatal care, nutrition guidance and glucose assessment could be arranged more closely. A more integrated pathway was seen as a direct way to reduce travel, repeated waiting and work disruption.

“I hope antenatal care, nutrition guidance and blood glucose assessment can be arranged on the same day as much as possible. That would save a lot of time. (P2)”

“What I need is clear dietary advice, online feedback on blood glucose and more integrated antenatal processes. (P13)”

## 4 Discussion

This study explored women’s experiences of time toxicity during GDM management and the perceived factors shaping these experiences. The findings showed that GDM care added a new timetable to pregnancy. Women described visible time costs related to hospital follow-up and less visible time costs related to daily self-management. Work demands and childcare significantly limit the time for GDM care. They also suggested ways to lighten this burden, such as providing a more specific guideline, providing online feedback to the participants, and incorporating outpatient service arrangements into the process.

The results are in line with the concept of time toxicity. In oncology, time toxicity refers to the time spent travelling, waiting, receiving care and managing care-related tasks at home (15). A recent qualitative study also showed that time burden can include both visible healthcare contact and less visible care work outside hospital (16). In this study, women with GDM did not usually experience long treatment sessions. Instead, time toxicity appeared through repeated tasks. These tasks included glucose monitoring, meal planning, postprandial activity, insulin use, information searching and repeated follow-up. This suggests that, in GDM, time toxicity may be expressed through recurrence, timing requirements and interruption of daily routines.

Daily self-management was an important source of time burden. Previous qualitative evidence has shown that women with GDM face barriers related to diet, exercise, glucose monitoring, education, family support and social context (17). Our findings are consistent with this evidence. However, the participants in this study also emphasised the time structure of these tasks. Daily self-management has become a major time-consuming burden. Blood glucose monitoring is fast, but breaks the day into a number of repetitive testing time points. It also takes longer to manage a diet, compared to selecting healthy foods. At nearly every meal, women must think about which foods will be eaten, whether they are fruits or staples, how they will fill them, and whether they might have an impact on blood sugar. Insulin-taking times must be coordinated with eating, insulin injection, post-eating, and testing for women taking insulin. This may also account for the fact that women know of the importance of self-management and that self-management of GDM is still challenging.

This repetitive scheduling had a strong correlation with the emotional stress experienced by the participants. Some of the females became nervous prior to undergoing the test, and others attempted to defend each of the abnormal readings by examining their diet or activities. These same issues of anxiety following diagnosis, feeling guilty about food, role conflicts, and concerns about insulin use were also identified in qualitative studies of gestational diabetes (18). Comprehensive thematic analysis also confirmed that women experiencing GD required support with psychological impact, information needs, lifestyle changes, social support, and care transition (19). This study further proposed a time-related explanation. Anxiety is not just associated with the diagnosis of GDM, but also with every meal, every blood sugar measurement, and the adjustment process.

Social roles of women are also a factor affecting the time of toxicity. It’s hard for working women to manage their blood sugar levels, walk, and test blood sugar at the same time. Women who have children should manage their gestational diabetes self-management sensibly, care for their children, and perform household chores. The practical help of the family to prepare appropriate food or to follow the meal of the person with the burden can help relieve this burden (20). Meanwhile, women may feel like they are being monitored or blamed with frequent reminders. This is similar to the results of the qualitative research, which suggests that social support plays a positive role in promoting self-management of GDM, but can be constrained by interpersonal and practical barriers (21). Thus, nursing assessment needs to also take into account their work schedules, parenting responsibilities, family eating habits, and support options.

Follow-up in hospital has resulted in a greater time burden. The processes of traveling, registration, waiting, examination, short consultations, and the interaction between the several departments were described by the participants. It will be difficult for women whose homes are not located near the hospital, as well as for pregnant women in their final trimester. Past studies have indicated that women with GDM are more likely to be instructed in clear recommendations and coordinated care when provided with dietary recommendations and SMBG support (22). Another qualitative study of prenatal care for diabetes also emphasised the potential to better inform mothers with more relevant lifestyle information and to rationally utilise the use of telemedicine to improve the quality of maternal medical services (23). Our research findings uphold these directions. Face-to-face visits are extremely important if blood sugar control is not well maintained or if there are changes to treatment plans. But if the primary purpose is to analyse blood sugar logs or to provide basic answers, then remote feedback could lower unneeded time on the road and waiting.

The findings have relevance for the nurse, midwife and diabetes educator. The educational information should be more specific than general advice, such as help in controlling diet, exercise and monitoring blood sugar. Women require examples of things that are close to their real-life events, e.g., meal combinations, take-out food choices, cafeteria food, fruit consumption, missed blood sugar checks and insulin administration times. Other services are also not flexible enough after the event has taken place. Low-risk issues and blood sugar record reviews can be done via structured online feedback, and women with poor blood sugar control or who require medication changes also require timely face-to-face care. The outpatient services can also be arranged closer. Having a prenatal exam, dietary advice, and checking blood sugar all on one day may minimise the inconvenience of frequent waiting periods, not coming to work, and arranging with family. Digital support design should be carefully considered and should aim to decrease the burden of decision-making, not to add reminders, information, or monitoring.

### 4.1 Limitations

Several limitations of this study should be recognised. Firstly, the findings of the study are from a tertiary hospital in the central part of China and therefore may not apply to the primary care experiences of pregnant women with GDM in rural or other medical system settings. Secondly, the sample size is small, acceptable in qualitative research, but this restricts generalisability of the research results. Thirdly, only women who experienced GDM were interviewed, while partners, family members, nurses, midwives, and doctors may offer additional viewpoints on time burdens generated and reduced. Fourth, the study did not measure the time spent on travel, wait times, or on monitoring and care coordination. A comprehensive analysis could be made in the future, using interviews and time diaries/service use. Fifth, women with GDM were not formally involved in the development of the interview guide, which may have limited the extent to which the guide reflected their priorities and perspectives. Future studies should consider involving women with GDM at an earlier stage of interview guide development, as member checking conducted after data analysis cannot fully replace patient involvement during the study design stage. Finally, the concept of time toxicity was originally developed in oncology. The word ‘toxicity’ may also carry different connotations across languages and pregnancy-related contexts. Its interpretation and acceptability in GDM care should therefore be examined in larger and more diverse populations, and patient-facing alternatives such as ‘time burden’ may warrant consideration.

## 5 Conclusions

This qualitative study aimed to look at the experiences of the women about the time experience during the management of GDM and what factors could affect this time experience. GDM care has altered the rhythm of pregnancy with an increase in repetitive activities during the day. The time burden is more visible in the hospital follow-ups, travel and wait, and more subtle in other areas like blood glucose monitoring, diet planning, etc., post-meal activities, insulin usage, and continuous monitoring of blood glucose levels. This burden can be compounded or ameliorated by work arrangements, parenting responsibilities, household chores, family involvement and service arrangements. There is a need for nurses, midwives and diabetes educators to offer practical advice for education, flexible follow-up plans, and better synchronisation of care provision. These measures can help women adopt the management of GDM as a part of their everyday life and decrease time pressure and repetitive decision-making.

## Data Availability

The raw data supporting the conclusions of this article will be made available by the authors, without undue reservation.
